# Book Notices and Reviews

**Published:** 1864-12

**Authors:** 


### BOOK NOTICES AND REVIEWS.

*Military Medical and Surgical Essays:* Prepared for the United States Sanitary Commission. Edited by Wm. A. Hammond, M. D., Surgeon-General U. S. Army, etc. Philadelphia: From the publishers, J. B. Lippincott & Co.—through S. C. Griggs & Co., 39 and 41 Lake Street, Chicago, Ills.

These papers were written by a number of the leading medical men of the present day, at the request of the U. S. Sanitary Commission, for the purpose of distributing them gratuitously among the medical army officers. Their merit was such as finally to make it advisable to bring them together in a permanent form and more easy of access. The result is this beautifully executed volume of above five hundred pages, divided into seventeen chapters, each treating of a different subject. Though especially designed for army surgeons, it is not the less useful or interesting to the physician in private practice. An enumeration of the authors and the subjects on which they write is a sufficient commendation of the work. These are as follows: Military Hygiene and Therapeutics, by Alfred Post, M. D., and Wm. H. VanBuren, M. D.; Control and Prevention of Infectious Diseases, by Elisha Harris, M. D.; Quinine as a Prophylactic against Malarious Diseases, by Wm. H. VanBuren, M. D.; Vaccination in Armies, by F. G. Smith, M. D., and Alfred Stille, M. D.; Rules for Preserving the Health of the Soldiers, by Wm. H. VanBuren, M. D.; Scurvy, by Wm. A. Hammond, M. D.; Miasmatic Fever, by John T. Metcalfe, M. D.; Continued Fever, by J. Baxter Upham, M. D.; Yellow Fever, by John T. Metcalfe, M. D.; Pneumonia, by Austin Flint, M. D.; Dysentery, by Alfred Stille, M. D.; Pain and Anæsthetics, by Valentine Mott, M. D.; Hemorrhage from Wounds, and the Best Means of Arresting it, by Valentine Mott, M. D.;

Treatment of Fractures in Military Surgery, by John H Packard, M. D. ; Amputations, by Stephen Smith, M. D. ; The Excision of Joints for Traumatic Causes, by R. M. Hodges, M. D. ; Venereal Diseases, by Freeman J. Bumstead, M. D.

---

*Outlines of Surgical Diagnosis :* By Geo. H. Macleod, M D., F. R. C. S. E., EEL, Fac. Phys. and Surg. Glasgow ; Lecturer on Surgery Anderson's University ; Surgeon to the Glasgow Royal Infirmary, and the Lock Hospital ; late Sen. Surgeon Civil Hospital, Smyrna, and General Hospital in Camp before Sebastopol. Memb. Cor. de la Soc. de Chir. de Paris ; and author of "Notes on the Surgery of the War in the Crimea." First American Edition. Reprinted from Advanced Sheets. New York : Bailliere Brothers, 520 Broadway.

In reviewing medical works it has been our object and desire to peruse them carefully, and without prejudice, determined not to be influenced by any preconceived ideas of our own that would in any way tend to bias the reader, causing him to diverge from the path of generally accepted facts. We think we have studied this part of a reviewer's duty on all occasions.

The work before us is one of great value and cannot fail to be of infinite service ; many of the articles, it is true, are brief, and more might have been written, but it is also true that what is written is lucid and comprehensive. The disease known as Anthrax or Carbuncle our author defines in a few words "as a circumscribed inflammation of the subcutaneous cellular tissue, leading to its death and expulsion." "It may arise as a simple affection or be an accompaniment of very serious constitutional ailments. It occurs in persons beyond middle life who are of a plethoric or feeble habit of body, or irritable constitutions, or who are intemperate in eating and drinking." It may occur in any part of the body, but is generally met with where there is cellular tissue rich in blood-vessels. It is especially on the posterior surface of the trunk that Anthrax occurs. It is rarely on the limbs."

We cannot resist quoting at length the author's language, as it illustrates the clearness of style and the easy manner he possesses of describing and contrasting diseases.

"Symptoms, a feeling of pricking, itching, or heat in the part is followed by redness, swelling, dark livid discoloration, and a throbbing, tensive, burning pain. The parts get brawny and shining. The tumor does not "point," but remains flat, though it may be as a whole elevated above the surface. There is a large circumscribed, deep-set base, having some little boils, at other times small turbid vesicles around its circumference. In time the surface gets honey-combed by small ulcerous openings, which exude a dirty, scanty, irritating, thin, fœtid pus, the amount of which may be increased by applying pressure to their neighborhood," etc., etc.

"The local affection is preceded or accompanied by constitutional symptoms of considerable severity, which appear rarely," etc., etc.

"The affections with which it is possible to confound Anthrax are, (a) Phlegmon; (b) Phlegmonous erysipelas (at its outset); (c) Malignant Pustule; (d) Boil; (e) Bedsore. The distinction is made by observing—

1. "The history of the rise and progress of the malady. In this way it is distinguished from b, c, and e."
2. "Large size from c and d."
3. "Age and disposition of person affected (a). Boils occur in the young and vigorous.
4. "Position. May occur on any part; but it is chiefly on the back that Anthrax is seen, and not especially on exposed parts, as (c); and is not especially on the parts most liable to pressure, as in (e).
5. "Not contagious," (b, c).
6. "Several small openings, (d, only one)."

"From common boil, its size, its not pointing, its occurring in old and feeble persons, its having many openings, being of a darker red, and having a deeper base, will distinguish it. Malignant pustule begins as a small pimple after inoculation. The local affection is the leading feature in the case, and the constitutional symptoms follow; while the reverse is the case in Anthrax.

This style is observable throughout the work—no superfluity of words—making the volume more welcome to the student. To Fractures and Dislocations the author has devoted many pages, elucidating the diagnostic characters of each form of injury, which are well worth special attention.

Gun-Shot Wounds; the entrance and exit of the ball; the size and shape of the wound, and the condition of the surrounding parts, are well described, notwithstanding but little is written on the subject.

The description of various tumors presenting themselves in different locations, especially those of the groin, cannot fail to impress the reader of the clear and succinct manner of the author's style.

In fact the entire work abounds in useful matter, and needs no further notice from us. It cannot fail to take a high rank among the standard works of our profession. We therefore recommend it to the student and practitioner.

---

*A Comprehensive Medical Dictionary;* Containing the pronunciation, etymology, and signification of the terms made use of in Medicine and the Kindred Sciences. With an Appendix, comprising a complete list of all the more important Articles of the Materia Medica, arranged according to their Medicinal properties. Also an explanation of the Latin terms and phrases occurring in Anatomy, Pharmacy, etc., together with the necessary directions for writing Latin Prescriptions, etc., etc. By J. Thomas, M. D., Author of the system of pronunciation in Lippincott's Pronouncing Gazetteer of the World. Philadelphia: J. B. Lippincott & Co. 1864.

The want of a work like the present has long been felt by the profession. Desirable as it might be that every physician should possess such a thorough knowledge of the foreign languages, that no extra aid should be required either for definition or pronunciation of any term occurring in Medical Science, yet the contrary fact is the rule. To supply this deficiency the work before us is offered to the profession. An analysis of its contents is unnecessary, as its objects are indicated in the title page. That the author has succeeded in the undertaking, none will doubt who are familiar with his former labors, contained in Lippincott's Pronouncing Gazetteer.

We heartily welcome any means which shall produce greater uniformity in pronunciation by members of the medical profession in this country. This work should effect the object. We therefore recommend it to every medical man for frequent reference.

---

*The Army Surgeon's Manual:* for the use of Medical Officers, Cadets, Chaplains, and Hospital Stewards, containing the Regulations of the Medical Department, all General Orders from the War Department, and Circulars from the Surgeon General's office, from January 1st, 1861, to July 1st, 1864. By William Grace, of Washington, D. C. Published by permission of the Surgeon General. New York: Bailliere Brothers, 520 Broadway. 1864.

This little volume of 200 pages, contains much information of interest to the *general* practitioner; and the rules, regulations and forms relating to the Medical Department of the Army, which renders it indispensible to the Army Surgeon.

---

*Slade on Diphtheria.*—This is the second and thoroughly revised edition of the Fiske Prize Essay upon Diphtheria. It contains the sum of reliable information relating to this formidable malady—its history, nature and treatment. The latter is indicated in the following brief extract: 'That it is now regarded as an asthenic disease, and consequently will bear no depletory measures, but on the contrary, requires tonics, stimulants and a nourishing diet, even in the early stages. Blisters, leeches and local bleeding of any sort should be prohibited." We do not hesitate to say that no physician should undertake the treatment of Diphtheria, who entertains opposite views. For sale by W. B. Kean & Co.

---

VACCINE MATTER.—Carefully selected Vaccine Matter may be obtained by enclosing One Dollar and addressing P. O. Drawer 5787, Chicago, Ills.

